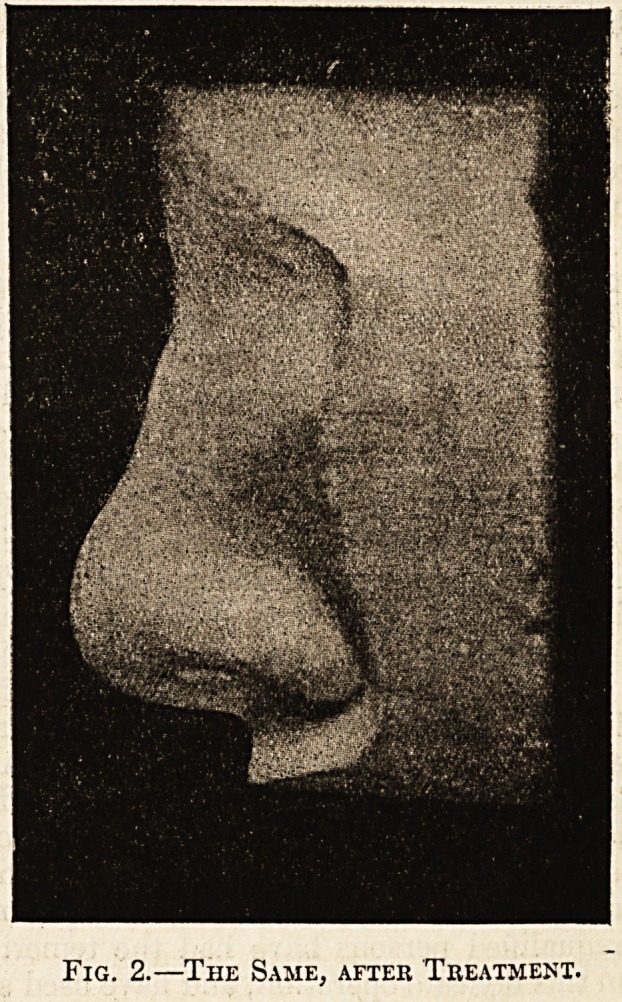# The Use of Paraffin in Scars and Other Facial Blemishes

**Published:** 1911-07-01

**Authors:** David Walsh

**Affiliations:** Senior Physician Western Skin Hospital, London, W.


### Hospital Clinics.

### THE USE OF PARAFFIN IN SCARS AND OTHER FACIAL BLEMISHES.

By DAVID WALSH, M.D. Edin., Senior Physician Western Skin Hospital, London, W.

For some reason that is not altogether apparent dermatologists have hitherto failed to avail themselves of recent advances in cosmetic surgery so far as the subcutaneous injection of paraffin is concerned. In the remedial treatment of exuberant scars, it is true, they have done a great deal of good work with such modern weapons as fibrolysin, $x$-rays, and the cataphoresis of various drugs. But the ordinary depressed and disfiguring scar they have for the most part left severely alone, in spite of the fact that more than ten years have elapsed since the introduction of paraffin as a method of alleviation, and at least five since the use of that material has been placed on the footing of a safe and scientific surgical procedure. This neglect is not altogether easy to understand when one realises the enormous extension of the study of diseases of the skin that has taken place during the present generation, and when one reflects that integrity of form is no less essential to the personal appearance and peace of mind of their patients than that of colour and other qualities of a wholesome skin. Be that as it may, it is now desired briefly to draw attention to the fact that various defects in skin contour, both natural and acquired, may be remedied by a process that in suitable cases and in skilled hands provides a safe, speedy, and effectual remedy.

Fig. 1—Dr. Spicer's Case of "Saddle Nose" before Injections.

There is no need to review the history of paraffin injections at length. The credit of the discovery is due to the German Gersuny, who published his first cases in 1899. At the same time it should be noted that the French claim priority for Delanger, of Tournai, who, earlier in the same year, injected paraffin experimentally under the skin of his own arm. The paraffin used originally by Gersuny was that known as German white vaseline, with a melting-point between 98°.8 F. and 104° F. After wards a harder paraffin was substituted, until at length the French employed one with a melting-point 141°.8 F.

It may here be stated that the melting-point is determined by the varying admixture of soft and hard paraffin; the softer the paraffin the lower the melting-point, and *vice versâ*. In either form, hard or soft, the material remains permanently in the tissues, where it gives rise to some amount of inflammatory reaction. Mouskowicz [1] says on this point that, like every foreign body, paraffin excites a reaction, though of a very mild type, in the surrounding tissues. In time granulations penetrate it, become organised, and form a meshwork containing paraffin. This sponge work becomes continually finer, until the mass of paraffin is converted into a kind of emulsion, which he believes is eventually capable of being absorbed by the lymphatics. That absorption is, however, improbable, is shown by some of Ger-

Fig. 2.—The Same, after Treatment.

suny's artificial injections remaining unaltered at the end of two years.

The early operators used paraffin melted and injected in a fluid condition. They found that the rapid cooling and setting of the paraffin gave rise to various difficulties, of which not the least lay in the blocking of the needle and the risk of burning the skin. These difficulties were met by improved apparatus and better technique, and many brilliant successes were recorded. In our own country the classical case was one of "saddle" nose moulded into comparatively good and presentable shape by Dr. Scanes Spicer,[2] and reported by him in April 1902. This was followed in December of the same year by a series of cases of a similar deformity operated upon by Mr. Stephen Paget.[3] By the kindness of Dr. Spicer two illustrations are here reproduced, showing the nose before and after treatment (figs. 1 and 2).

In the year 1904, however, a great advance was made by Dr. Lagarde, of Paris, who showed that it is possible to force cold paraffin into the tissues under pressure. His method was announced to the Académie de Médecine in March 1904. In 1907 he published a fuller description in a book under the title "La Prothèse par les injections de Paraffine." In that work he gave an exhaustive historical and contemporary review of the subject. Various syringes were figured, including his own, for the injection of cold paraffin under pressure (*sous pression*).

A note in Dr. Lagarde's list of references gives, under his own name, the following: "Technique et instrumentation nouvelles pour faire les injections de paraffine à froid. Communications faites à l'Académie de médecine le 1er Mars 1904, à la société médicale des hôpitaux le 12 Mars 1904, et à la société de médecine de Paris, le 23 Mars 1904."

The best paraffin for the purpose is, in my opinion, one with a melting-point of about 105° F. Theoretically it might become fluid should the temperature of the recipient's body rise above that particular point, but in practice it is found that no such untoward result follows when the thermometer has registered a fever temperature above 105° F. A vital point is the absolute sterilisation of the paraffin, a condition that can be obtained only by prolonged treatment in skilled hands.[4] Neglect of this point has led to disastrous consequences in cases where medically unqualified persons have had the temerity to perform this delicate operation, and have used septic instruments or have neglected some one or more of the details necessary to the perfect asepsis. Needless to say the utmost care is needed in every step of the process. Instruments must be boiled, the paraffin melted in a water-bath, and the skin carefully sterilised. The needle is introduced into the skin about half an inch from the spot to be filled up with the paraffin. Pressure is then applied to the piston rod, and the paraffin when forced into the tissues is instantaneously moulded into the desired shape by the fingers of the operator or an assistant. Where the skin is loose an ordinary serum syringe may answer the purpose, but where there is any tension to overcome it is necessary to use one of the special instruments devised to apply the necessary pressure. In any case the operator should rehearse everything beforehand, or he will find himself faced with various unexpected difficulties at the time of operation. The puncture left by the needle can be sealed with a little collodion or treated with an antiseptic lotion and powder. It is better to inject too little than too much paraffin, and to fill up a large cavity by injecting small quantities at intervals of a few days. Should there be too large a quantity it can be removed only by cutting it out, a fact that it is well to bear in mind when a patient wishes to have more paraffin injected into a given spot than appears expedient to the operator.

Accidents are almost unknown when the operation is properly conducted. The risk of injury to veins is slight if a due attention be paid to the chief facts of anatomy. If there be any doubt the needle may first be inserted into the tissues, when the puncture of a vein will be registered by a flow of blood.

For ordinary small operations about the face no anæsthetic, local or general, is needed. The only pain is that caused by the trifling puncture. Where the tissues are tight and much paraffin has to be injected, as in building up a nose, it may be necessary to have the patient under the influence of chloroform.

As to the conditions that can be relieved by this method, that of the sunken nose is one of the most striking. There can be no more repulsive and humiliating defect in the human face than a gross defect of the nasal organ, especially as nine persons out of ten attribute the disease to syphilis.

Atrophy of the face due to various palsies can be remedied by the paraffin method. That fact has been well illustrated by pictures of a patient before and after treatment published by Mouskowicz.[5] The change effected in the face by this simple and safe cosmetic operation can only be realised from an inspection of the photographs in question.

A remarkable case is noticed in the *Medical Review*.[6] The patient suffered from bilateral facial atrophy, and the hollows of the cheeks and temples were successfully filled up with paraffin by Dr. B. T. Burley, of Boston, in nine sittings.

In some cases it may be desirable to obliterate deep lines or wrinkles from a face. A good instance is the two parallel lines sometimes seen between the eyebrows, the effect of which is to convey the appearance of a permanent frown. This may appear in youth or early adult life, but whatever the time of its onset it may be safely and permanently removed by the timely injection of a small quantity of paraffin. Obviously it would be difficult to say to what extent the operator would be justified in attempting to obliterate ordinary wrinkles, but there are certain exceptional and disfiguring lines in many faces, especially those occurring in early adult and middle life, that there need be no hesitation at all in attempting to remove or modify.

One of the most useful applications is in those disfiguring cases of depressed scar sometimes seen over the site of a former "tooth" abscess or bone.

In rare instances it may be necessary to free adhesions by cutting them through with a narrow-bladed tenotomy knife. The scars resulting from glandular abscess are often deeply depressed and unsightly, and these can be readily filled with paraffin.

The scars left by small-pox and scar-leaving acne can be speedily and effectually filled up, unless, indeed, they be confluent and involve the whole surface. The field opened up in this direction alone is worthy the attention of every medical practitioner. The depressed scars left by traumatism, by tubercular and syphilitic gummatous growths; in short, by any lesion involving loss of subcutaneous tissue substance, may be more or less favourably modified by the judicious use of paraffin in the filling up of gaps and the levelling of surfaces.

In conclusion, it may be said that the introduction of the treatment of skin deformities by the injection of cold paraffin has opened up a new and not unimportant chapter in cosmetic surgery. In practice it is applicable to a number of distressing and disfiguring defects arising from a wide variety of causes with an assurance of safety and certainty that is by no means associated with the majority of our therapeutic measures.

It is interesting to note that the use of paraffin has been advocated in the identification of criminals. The methods now in use, although reasonably sure, are none the less tedious and complicated. Dr. Icard, in the *Archives d'Anthropologie Criminelle*, has recently described a plan for which he claims simplicity and permanency. His method is to inject paraffin into various parts of the body of the criminal on a system so arranged as to indicate the nature and the quality of his offence. Thus he suggests that "for example, it might be convenient to choose the inner edge of the right shoulder-blade to mark professional thieves. The limited area could then be divided into three parts: the upper for the most dangerous thieves, the middle for the average, and the lower for the least dangerous." The police would then be able to learn a good deal about a suspected person by running a finger down his right shoulder-blade. The paraffin would form a permanent solid lump under the skin; or, if cut out—the only way by which it could be removed—its former site would be indicated by a tell-tale scar.

---

[1] Dr. Ludwig Mouskowicz, "Paraffin in Surgery," *Wien. Klin. Woch*, June 20, 1901, p. 603.
[2] *Clinical Journal*, April 9, 1902.
[3] *Lancet*, December 20, 1902, p. 1694.
[4] A scientifically prepared paraffin can be obtained of Mr. F. A. Rogers, Pharmaceutical Chemist, 327 Oxford Street, London, W.
[5] *Wien. Klin. Woch.* Jan 8, p. 33, 1903. See also *Medical Review*, vol. vi. p. 211.
[6] *Medical Review*, 1904, p. 372.

## Figures and Tables

**Fig. 1 f1:**
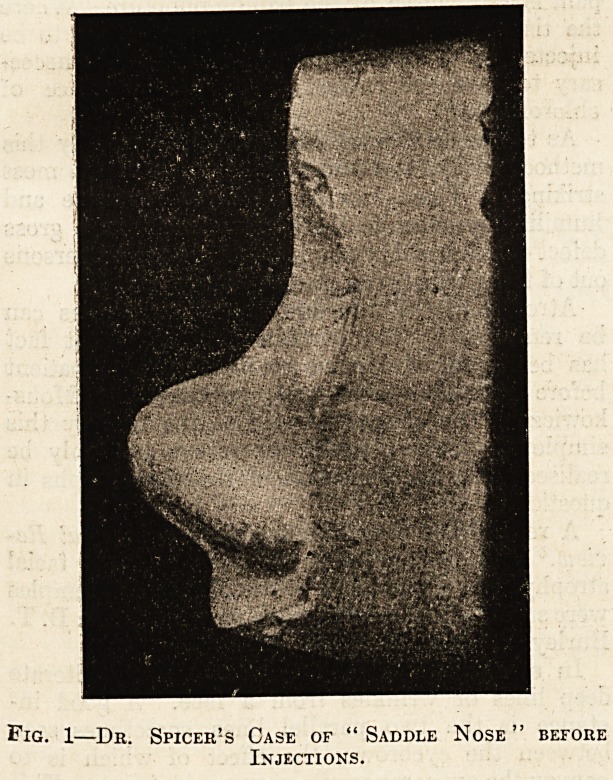


**Fig. 2 f2:**